# La tumeur de Buschke-Löwenstein

**DOI:** 10.11604/pamj.2013.14.94.2463

**Published:** 2013-03-10

**Authors:** Fadwa El Amrani, Badredine Hassam

**Affiliations:** 1 Service de Dermatologie, CHU Ibn Sina, Université Med V, Souissi, Rabat, Maroc

**Keywords:** Buschke-Löwenstein, tumeur, condylome acuminé géant, papillomavirus humain, Buschke-Löwenstein, tumor, Giant condyloma acuminatum, human papillomavirus

## Image en médicine

La tumeur de Buschke-Löwenstein, ou condylome acuminé géant, est une maladie sexuellement transmissible à papillomavirus humain, le plus souvent de type 6 et/ou 11. Elle est rare et toujours précédée de condylomes acuminés. Cette tumeur épithéliale a des rapports encore mal définis avec le carcinome verruqueux, l’aspect histologique est en fait bénin, quoique son aspect clinique évoque plutôt le contraire. Elle affecte particulièrement l’homme et se caractérise essentiellement par son extension en profondeur, son potentiel dégénératif et son caractère récidivant après traitement. La chirurgie constitue le traitement de choix. Nous rapportons le cas d’un patient âgé de 45 ans, ayant comme antécédent une notion de vagabondage sexuel, qui consulte pour une tumeur périnéo-scrotale évoluant depuis 15 ans. L’examen clinique notait la présence d’une lésion tumorale infiltrée, papillomateuse en chou-fleur, ano-périnéale et scrotale, fétide et indolore. Les aires ganglionnaires étaient libres. Les sérologies VIH, syphilitique et des hépatites B et C étaient négatives. L’examen histologique d’un prélèvement biopsique a mis en évidence une hyperplasie épithéliomateuse qui a été faite d’un revêtement malpighien acanthosique, papillomateux, surmonté par une hyperkératose parakératosique avec présence de koïlocytes signant l’infection par le virus HPV (Papillomavirus), il n’a pas été noté d’atypie cellulaire. Le diagnostic de tumeur de Buschke-Löwenstein a été retenu et le patient a été adressé au service de chirurgie urologique où une exérèse large a été réalisée. Il n’a pas été noté de récidive avec un recul de 3 ans.

**Figure 1:**
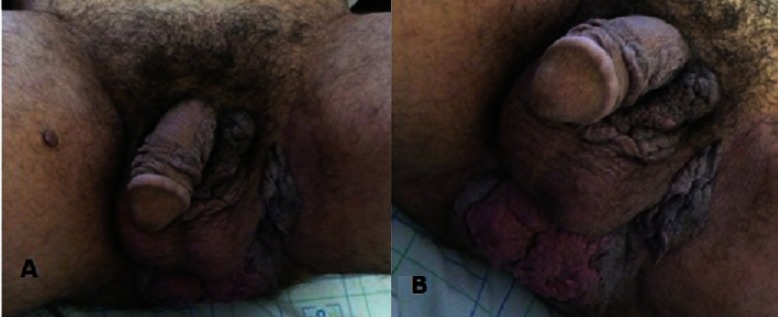
Tumeur papillomateuse en chou-fleur périnéo-anale et scrotale A: vue antérieure, B: extension périnéo-anale

